# Evaluation of Glandular Liposculpture as a Single Treatment for Grades I and II Gynaecomastia

**DOI:** 10.1007/s00266-018-1118-x

**Published:** 2018-03-16

**Authors:** Islam Abdelrahman, Ingrid Steinvall, Bassem Mossaad, Folke Sjoberg, Moustafa Elmasry

**Affiliations:** 1 Department of Hand and Plastic Surgery, Burn Centre, Linköping University Hospital, 58185 Linköping, Sweden; 2 Department of Clinical and Experimental Medicine, Linköping University, Linköping, Sweden; 3 Plastic Surgery Unit, Surgery Department, Suez Canal University, Ismailia, Egypt; 4 Department of Anaesthesiology and Intensive Care, Linköping University, Linköping, Sweden

**Keywords:** Gynaecomastia, Liposculpture, Liposuction, Patient satisfaction

## Abstract

**Background:**

Gynaecomastia is a benign enlargement of the male breast, of which the psychological burden on the patient can be considerable, with the increased risk of disorders such as depression, anxiety, and social phobia. Minimal scarring can be achieved by liposuction alone, though it is known to have a limited effect on the dense glandular and fibroconnective tissues. We know of few studies published on “liposuction alone”, so we designed this study to evaluate the outcome of combining liposuction with glandular liposculpturing through two axillary incisions as a single treatment for the management of grades I and II gynaecomastia.

**Methods:**

We made a retrospective analysis of 18 patients with grade I or II gynaecomastia who were operated on by combined liposuction and glandular liposculpturing using a fat disruptor cannula, without glandular excision, during the period 2014–2016. Patient satisfaction was assessed using the Breast Evaluation Questionnaire (BEQ), which is a 5-point Likert scale (1 = very dissatisfied; 2 = dissatisfied; 3 = neither; 4 = satisfied; 5 = very satisfied). The post-operative aesthetic appearance of the chest was evaluated by five independent observers on a scale from 1 to 5 (5 = considerable improvement).

**Results:**

The patient mean (SD) overall satisfaction score was 4.7 (0.7), in which 92% of the responders were “satisfied” to “very satisfied”. The mean (SD) BEQ for all questions answered increased from 2.1 (0.2) “dissatisfied” preoperatively to 4.1 (0.2) “satisfied” post-operatively. The observers’ mean (SD) rate for the improvement in the shape of the front chest wall was 4.1 (0.7). No haematomas were recorded, one patient developed a wound infection, and two patients complained of remnants of tissue. The median (IQR) body mass index was 27.4 (26.7–29.4), 11 patients had gynaecomastia grade I, and 7 patients grade II. The median (IQR) volume of aspirated fat was 700 ml (650–800), operating time was 67 (65–75) minutes, 14 patients had general anaesthesia, and hospital charges were US$ 538 (481–594).

**Conclusions:**

Combined liposuction and liposculpturing using the fat disruptor cannula resulted in satisfied patients and acceptable outcomes according to the observers’ ratings. It could be a useful alternative with an outcome that corresponds to that of more expensive methods.

**Level of Evidence IV:**

This journal requires that authors assign a level of evidence to each article. For a full description of these Evidence-Based Medicine ratings, please refer to the Table of Contents or the online Instructions to Authors www.springer.com/00266.

## Introduction

Gynaecomastia is benign enlargement of the male breast, of which it is the most common disorder, with a reported incidence of 36% [1]. Pathological causes include taking drugs, relative or absolute excess of oestrogen, decrease in circulating androgens, or no clear cause (idiopathic). There is a benign proliferation of the glandular tissue, unlike pseudogynaecomastia in which the enlargement is merely the result of obesity and deposition of fat [2]. Regardless of the type of gynaecomastia, if it persists for more than a year the breast tissue will become more fibrous and resistant to medical treatment. At this stage, resection is the mainstay of management [3].

The psychological burden of gynaecomastia on the patients can be appreciable, making them at increased risk of psychological disorders such as depression, anxiety, and social phobia [4, 5]. This necessitates intervention in most cases to restore the masculine look of the chest and achieve psychological satisfaction, particularly in grades I and II gynaecomastia [6] in which excision of skin is seldom required. The presence of unsightly scars detracts from the success of the operation, despite the efficient reduction in breast volume and the skin envelope. Minimal scarring can be achieved by liposuction alone. Although liposuction is known to have a limited effect on the dense glandular and fibroconnective tissues [7], these tissues tend to be infiltrated by enough fat for the liposuction cannula to be able to penetrate, to reduce the projection in the subareolar area, and to create a normal-looking chest wall [8] with a dramatic retraction of the skin envelope that obviates the need for its excision [7]. Recently, new types of cannulas have been introduced including fat disruptor cannulas on which the edges of the openings are barbed to improve efficient breakdown and liposculpturing of the dense glandular tissue.

We know of few studies [7, 9–15] published on liposuction alone for correction of gynaecomastia. We designed this study to evaluate the outcome (patients’ satisfaction and assessment by an independent observer) of combining liposuction with glandular liposculpturing through two axillary incisions as a single treatment for grades I and II gynaecomastia.

## Methods

The records of all those (18 patients, 36 breasts) who presented with grade I or II gynaecomastia and were operated on with combined liposuction and liposculpturing without excision of glandular tissue during the period 2014–2016 were analysed retrospectively. All patients were treated by two surgeons (authors 1 and 3) at Ismailia Specialized University Hospital, Egypt.

Ethics approval for the study was obtained from the ethics and research committee at the Suez Canal University. Patients with secondary gynaecomastia, grades III or IV gynaecomastia, those treated by a technique other than liposuction, or those who refused combined liposuction and liposculpturing (which included the possibility of revision surgery), were not included in the study.

The patients’ age, patient’s body mass index (BMI), chronic illness, grade of gynaecomastia, preoperative breast asymmetry were recorded, as well as type of anaesthesia, total operating time, aspiration time, volume of fluid infiltrated, volume of fat aspirated, hospital stay (hours), days off work after the operation, and charges (in US$). Post-operative complications such as haematoma, infection, and seroma were recorded at the follow-up visits.

### Preoperative Preparation

All patients had their breasts examined for consistency (glandular, fat, or mixed), the position of the nipple–areola complex in relation to the inframammary line, and the symmetry of the breast. Secondary causes for gynaecomastia such as drugs or testicular tumours were excluded by clinical examination and hormonal blood assay for testosterone and oestrogen.

Each patient gave signed informed consent accepting combined liposuction and liposculpturing (which may include possible secondary revision to remove remnants of tissue), possible complications of anaesthesia, and possible complications particular to liposuction, mainly haematoma, asymmetry and irregularities of the breast, and having medical photographs taken before and after operation.

Complete blood pictures and coagulation profiles were requested for all patients who had the operation, either under local or general anaesthesia.

### Surgical Technique

The infiltrate was prepared for those who had local anaesthesia (0.5 l saline + 1 mg 1/1000 adrenaline + 2% lidocaine 12.5 ml). We added 8.4% sodium bicarbonate 7.5 ml to decrease the painful sensation during infiltration. For general anaesthesia, we used intravenous sedation and a laryngeal mask, as the whole procedure took about an hour. A stab incision 5 mm long was made 0.5–1 cm posterior to the anterior axillary line at the level of the sternal angle where the infiltration process was begun using a 3-mm straight blunt cannula with 20 holes, and the amount of fluid infiltrated ranged between 400 and 750 ml according to the size of each breast. The two breasts were infiltrated sequentially. We waited 15–20 min after infiltration to start the aspiration.

**Fig. 1:**
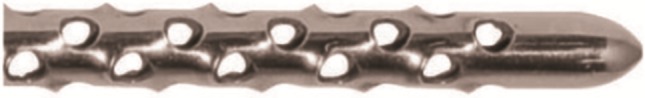
Fat disruptor cannula with multiple holes and barbed edges that was used for glandular liposculpture Reprinted with permission of Black & Black™

We used suction-assisted lipectomy in all cases. Tissue was aspirated from two openings, the original stab made for infiltration, and another 10 cm inferior to the first, just posterior to the anterior axillary line. This enabled “criss-cross” liposuction to achieve a smooth and even contour. A 5-mm blunt cannula with a Mercedes tip was used initially in the deep plane, followed by a 4-mm cannula to treat skin irregularities. The glandular tissue was treated with a fat disruptor, which is a cannula 36 cm long with multiple holes 4 mm in size and barbed edges (Black & Black Surgical™) (Fig. 1). This facilitated the breakdown of the tough glandular tissue, particularly in the retroareolar area, keeping in mind the need to spare 1 cm or more of thickness to avoid inversion of the nipple–areola complex. The end point was when an even contour had been achieved. Symmetry of the breasts was assessed primarily by bilateral pinch tests, as well as the duration of treatment for each breast. The openings were left open for free drainage, and no drains were inserted. A compression bandage was applied around the chest for 4 days, followed by a liposuction garment for 6 weeks, which the patient could take it off only while having a shower. The operation time in all cases did not exceed 80 min, and according to the international guidelines operations shorter than 120 min do not require thromboembolism prophylaxis. The risk factors associated with these patients were minimal; all the patients were young and were not overweight.

All patients were instructed to massage the two breasts frequently, starting from the first post-operative day, and they were encouraged to resume their regular physical exercise after 2 weeks. Time off work varied among patients according to the nature of their work, but 3 days were recommended. The first follow-up visit was usually on post-operative day four, mainly to exclude the presence of haematoma. Other complications such as infection or seroma were sought at the subsequent visit (1 week later).

### Patients’ Self-Reported Assessment

We used the data extracted from the Breast Evaluation Questionnaire (BEQ) [16] (which has previously been used to evaluate results after gynaecomastia [9]) to measure patient satisfaction. It is sent regularly to all patients operated on for gynaecomastia 6 months post-operatively, regardless of the type of operation done, as feedback for the clinic.

The questionnaire is divided into four parts: the degree of comfort with breast/chest size in different settings (intimate, social, and professional); the degree of comfort with appearance of breast/chest, dressed and undressed, in different settings (alone, presence of partner, other men, women, and healthcare professionals); the respondent’s satisfaction for himself and his partner; and the degree of satisfaction about specific features such as symmetry, numbness, and scars. The patients were asked to respond to all questions using a 5-point Likert scale (1 = very dissatisfied; 2 = dissatisfied; 3 = neither; 4 = satisfied; 5 = very satisfied). The significance of the differences between the scores before and after the operation was analysed with the Wilcoxon matched pairs test. Probabilities of less than 0.05 were accepted as significant.

### Observers’ Reported Assessments

Five independent plastic surgical consultants who were unaware of which operation the patient had had gave their opinions of three photographs (anteroposterior, oblique, and lateral) taken before and after the operation and 6 months apart. They assessed the following items: the improvement in the shape of the front chest wall on a scale from 1 (no improvement) to 5 (significant improvement), asymmetry of the breast, shape and projection of the nipple, and need for further procedures (Appendix). The results of the improvement in the shape of the front chest wall were presented as mean (SD) calculated on the ratings of the five observers. The categorical items of the assessment questionnaire were calculated on their frequency (number observed/total observations) as answered by all the observers. The level of agreement was analysed with Kendall’s coefficient of concordance [17], which is a statistical test for ordinal data to establish the extent of agreement between two or more judges beyond that which would be expected by chance alone.

### Observers’ Education

All the observers were consultants in plastic and reconstructive surgery who trained for more than 10 years in different hospitals either in Linkoping or Suez Canal universities. All of them were familiar with different techniques of gynaecomastia, and none of them took part of any of the operations.

## Results

**Fig. 2:**
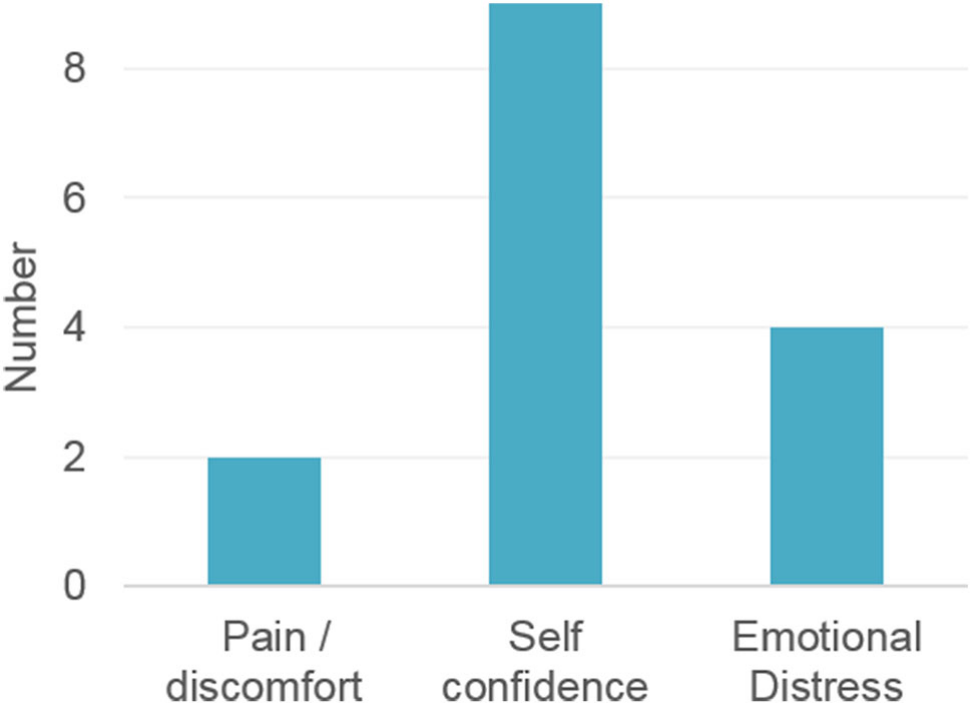
Reason(s) for surgery

Eighteen patients were operated on, two-thirds of whom had grade 1 gynaecomastia (Table 1). Two of the patients had a BMI more than 30. One of the patients was diabetic, but other than that none had chronic medical conditions. All patients were discharged on the day of operation, with a mean (SD) hospital stay of 6.6 (2.1) h. The mean (SD) ratio of infiltrated fluid–aspirated fat was 1.9 (0.3). One patient developed a wound infection and two complained of remnants of breast tissue and asked for a revision operation. The main reason for the surgery was self-confidence, followed by emotional distress (Fig. 2).

**Table 1:** Description of the patients and clinical data

No. of patients	18
Body mass index	27.4 (26.7–29.4)
Age (years)	31 (28–34)
Gynaecomastia grade I	11 (61)
Gynaecomastia grade II	7 (39)
Preoperation breast asymmetry	4 (22)
General anaesthesia	14 (78)
Total operative time (min)	67 (65–75)
Aspiration time (min)	45 (40–45)
Volume of infiltrated fluid (ml)	1375 (1200–1500)
Volume of aspirated fat (ml)	700 (650–800)
Duration of hospital stay (h)	8 (5–8)
Days off after the operation	3 (3–4)
Charges (US$)	538 (481–594)

Data are presented as median and 25th and 75th centiles or *n* (%)

The patients’ mean (SD) overall satisfaction score was 4.7 (0.7), in which most of the responders (92%) were satisfied or very satisfied, while the rest were neither satisfied nor dissatisfied (response rate 12/18).

**Fig. 3:**
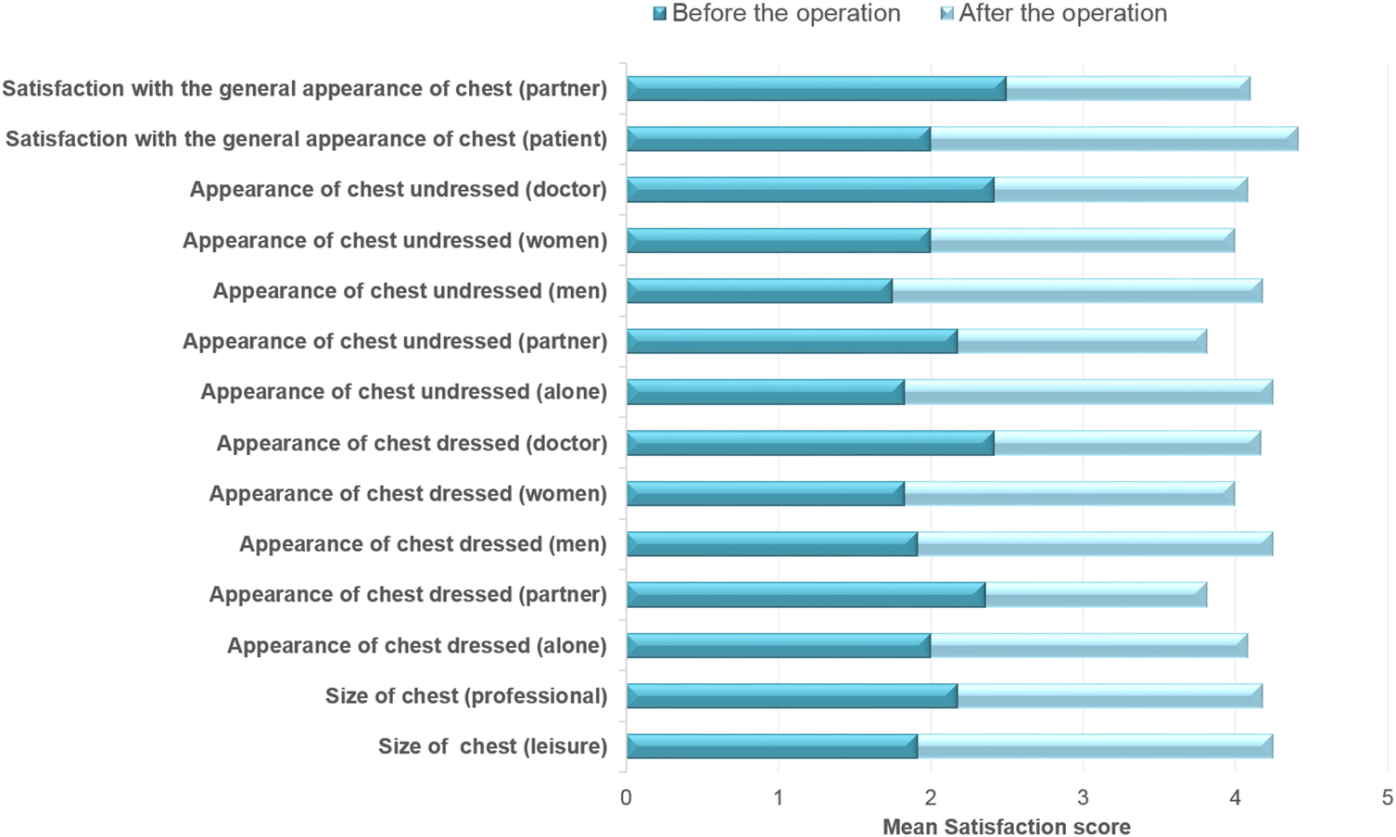
Patient satisfaction before and after the operation, assessed by the Breast Evaluation Questionnaire

The mean (SD) BEQ for all variables increased from “dissatisfied” 2.1 (0.2) preoperatively to “satisfied” 4.1 (0.2) post-operatively (*p* = 0.001). The mean (SD) increase in BEQ was 2.0 (0.3), and the biggest difference (2.4) was found in the three items: appearance of the chest undressed in the presence of other men; alone; and their own satisfaction with the general appearance of the chest. The least observed difference (1.5) was in the appearance of the chest dressed in the presence of the partner (Fig. 3).

**Fig. 4:**
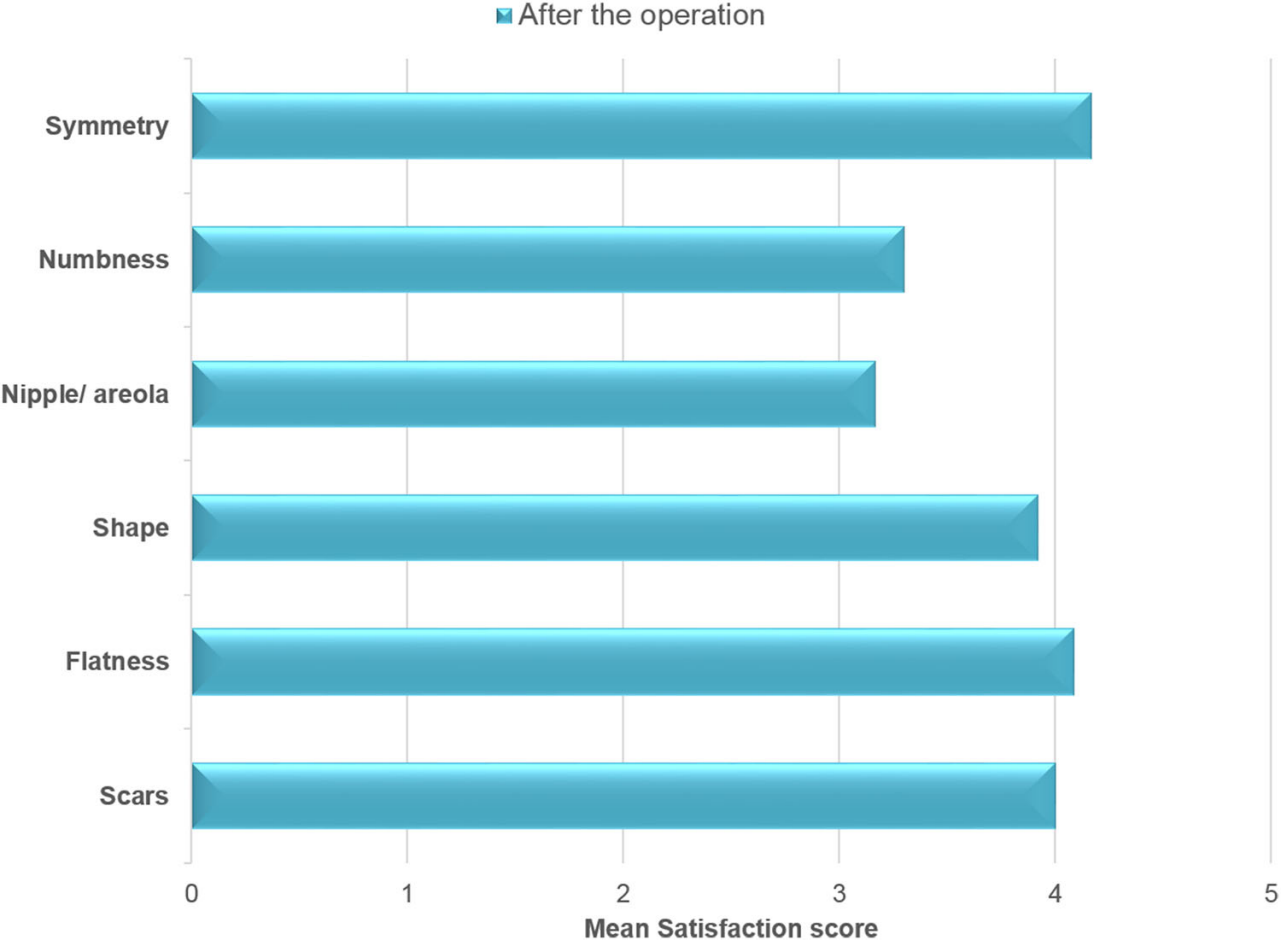
Patient satisfaction with specific chest features post-operatively

**Fig. 5:**
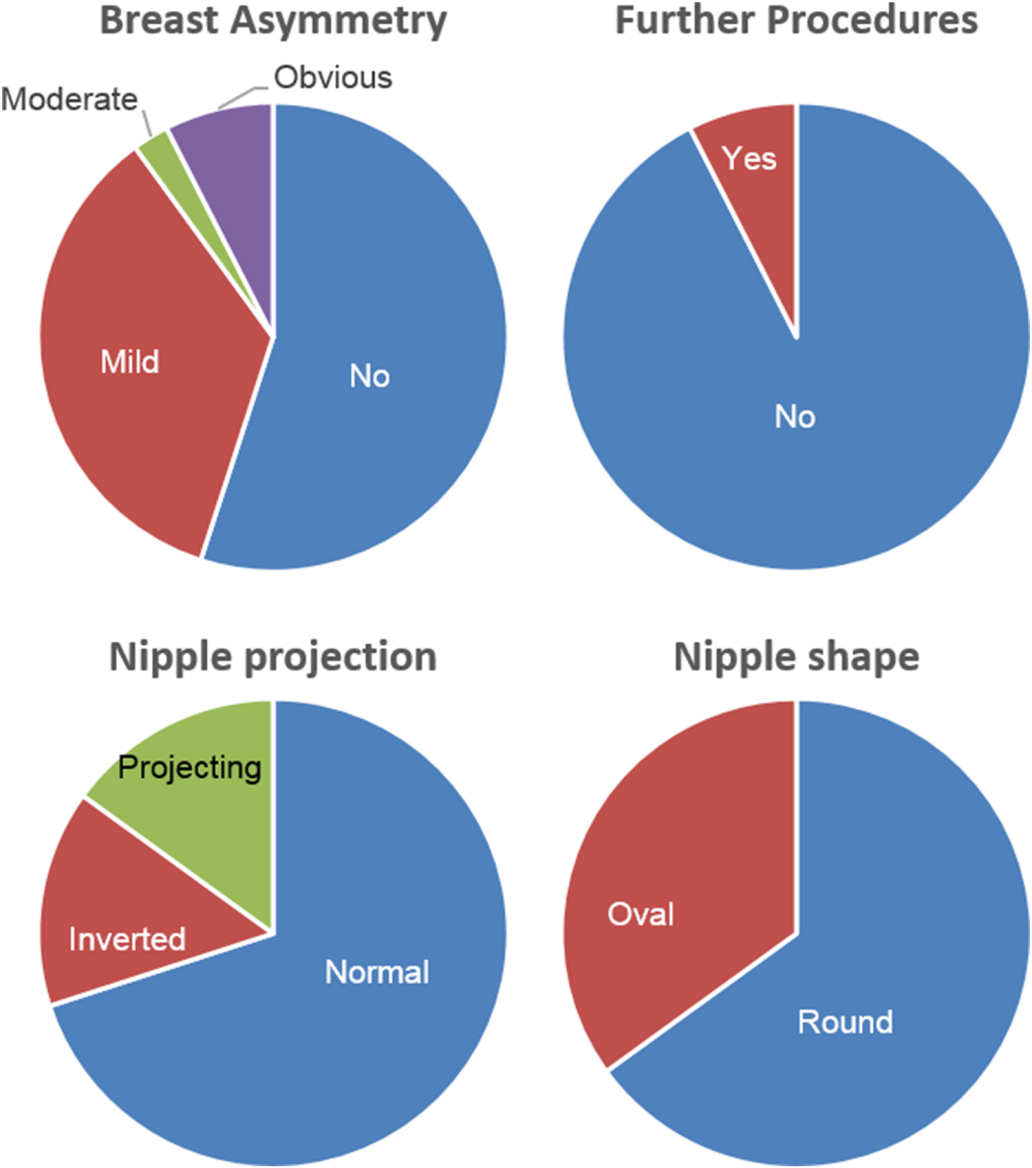
Observers reported assessment, percentage calculated on the total number of ratings

The number of responders who were satisfied or very satisfied with scars, flatness, shape of the breasts, and symmetry was 10/12 (Fig. 4). In the observers’ assessment, breast symmetry was achieved in 55% and mild asymmetry in 35% of the total ratings (Fig. 5).

Observers’ mean (SD) rate for the improvement in the shape of the front chest wall was 4.1 (0.7), where the patient with the lowest mean (SD) value had 3.2 (1.1) and the one with the highest 5.0 (0). According to the observers’ assessment, an acceptable post-operative result was achieved in 92% of the ratings while another session of liposuction procedure was suggested in 8% of the ratings (Fig. 5). The level of agreement (Kendall’s coefficient of concordance) was 0.61 (*p* = 0.003) for the improvement in the score of the shape of the front chest wall, 0.66 (*p* = 0.002) for asymmetry of the breast, and 0.58 (*p* = 0.005) for projection of the nipple.

**Fig. 6:**
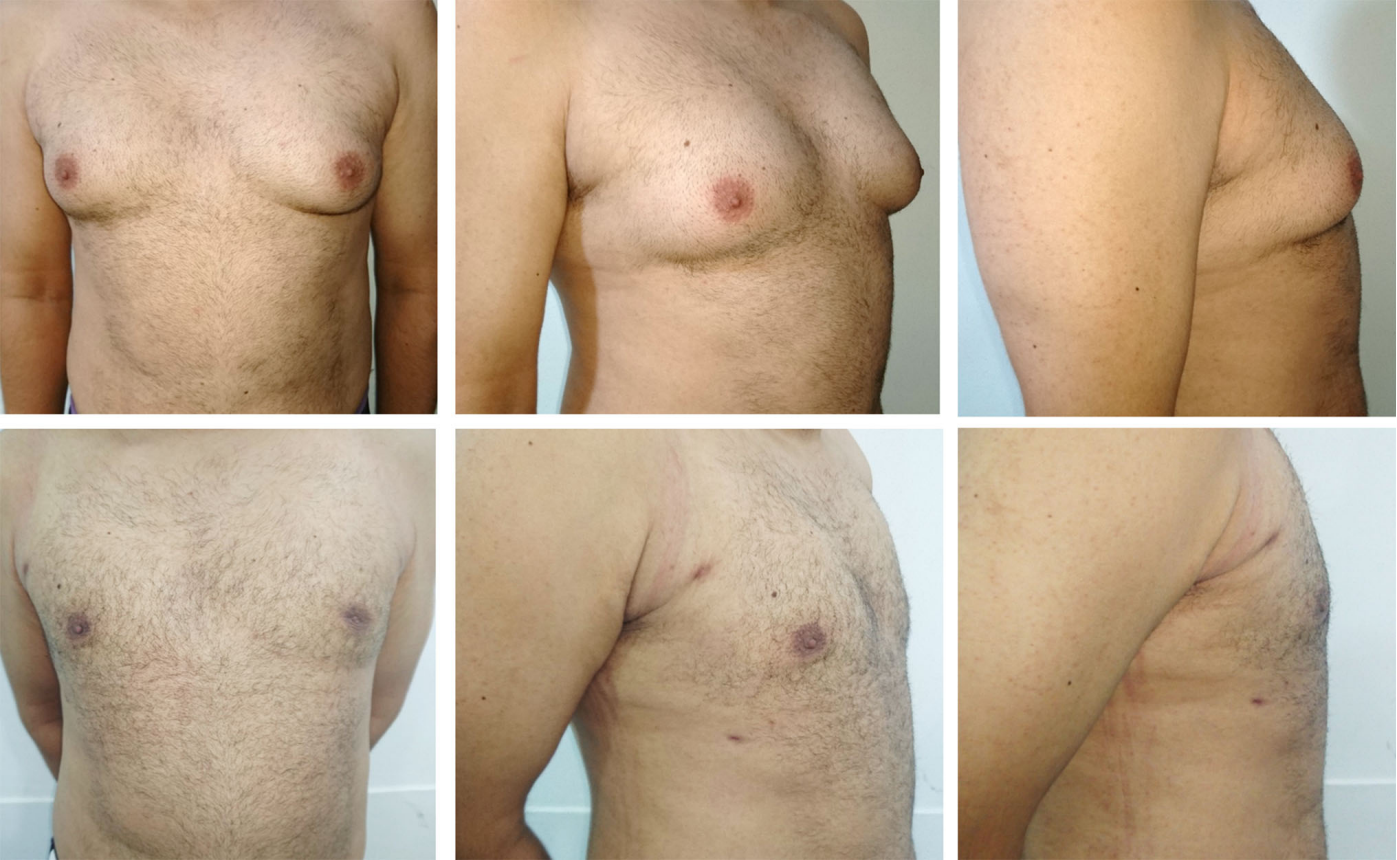
A 34-year-old patient 169 cm tall, who weighted 79 kg and had a BMI of 28, had bilateral gynaecomastia grade IIB. Upper row shows preoperative photographs with moderate enlargement of the breast tissue more prominent in the left breast; lower row shows post-operative results (6 months) with a satisfactory flat chest and masculine contour

**Fig. 7:**
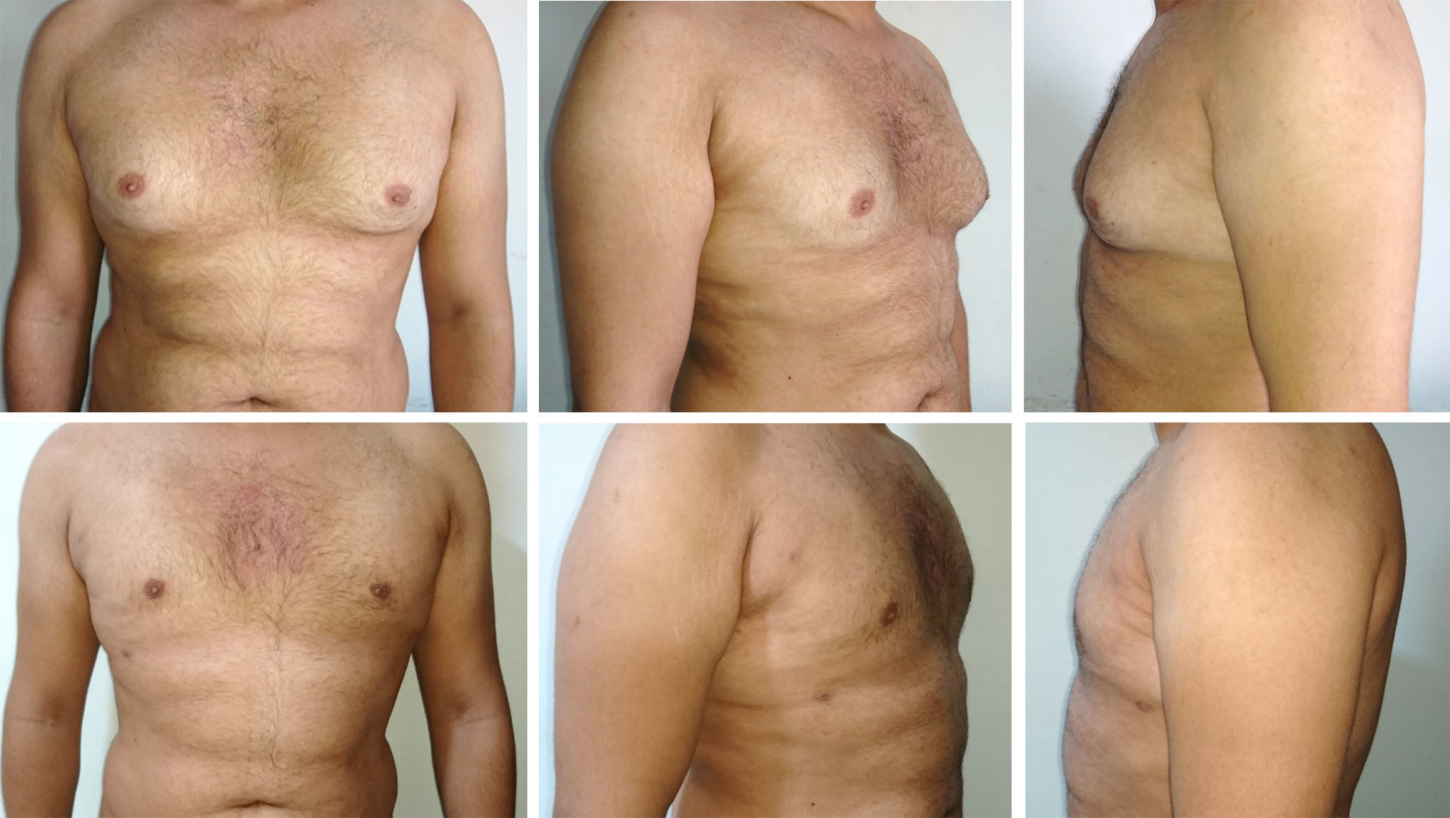
A 31-year-old patient 180 cm tall, who weighed 86 kg and had a BMI of 27, had bilateral gynaecomastia grade I. Upper row shows preoperative photographs with mild enlargement of the breast tissue; lower row shows post-operative results (6 months) with a satisfactory flat chest

Two examples of preoperative and post-operative photographs for patients who had gynaecomastia dealt with using liposuction and liposculpturing are presented in Figs. 6 and 7.

## Discussion

The satisfaction of patients with the result of the operation is the ultimate goal, with minimal scarring and reasonable charges. We considered that combined liposuction and liposculpturing would be suitable for patients with low-grade gynaecomastia (I and II). We evaluated the outcome in two ways: the BEQ, and the surgical observers’ assessment to strengthen the credibility of the results. The results of both arms showed similar trends with acceptable post-operative results.

Previous publications that have assessed patient satisfaction after treatment of gynaecomastia used different scales that assessed five or six items, most of which were related to the physical appearance of the breast [12, 18], or by measuring the overall satisfaction by means of yes/no answers [19–21]. The BEQ is more versatile and has been validated in general breast surgery [16] and gynaecomastia [9]. It assesses patient satisfaction in a more robust way than that in most previously published studies. It is also more comprehensive and improves the quality of the information available about outcomes.

The mean increase in patient satisfaction was greater in our study than in a published report [9] in which the same assessment tool was used, which could be explained in different ways: the difference in how consent was obtained from patients, including preoperative discussions about their expectations, the surgical technique, and the nature and social background of the patients.

The ideal surgical approach to manage gynaecomastia is to remove excess breast tissue, both glandular and fatty, and eliminate redundant skin, with minimal or no scarring. This achieves a good aesthetic outcome and is applicable to all grades of gynaecomastia. Despite the many surgical approaches and techniques proposed [6, 8, 22–28], this ideal approach has yet to be discovered. Since Teimourian and Perlman [29] described conventional liposuction combined with glandular excision for the treatment of gynaecomastia in 1983 the concept has become widely accepted, because of the difficulties of removing the tough glandular tissue by liposuction alone. Other authors, however, have contended that all grades of gynaecomastia could be treated by liposuction alone [13], in which a special cannula 2.3 mm long was recommended to remove breast tissues more easily [14]. Following Rosenberg’s lead, others have used special cutting gynaecomastia cannulas, such as a cut cannula with a sharp opening [15], or a biopsy punch [30]. Those cannulas, however, are more traumatic to both vessels and nerves, which led us to question a preference over the conventional methods.

In the late 1980s, Zocchi [31] developed ultrasound-assisted liposuction. During the last decade, many plastic surgeons changed from the traditional liposuction technique (suction-assisted, power-assisted, laser-assisted, and radiofrequency liposuction) to ultrasound-assisted liposuction.

Ultrasound-assisted liposuction turns electric energy into vibrations and causes thermal, cavitational, and mechanical effects that lead fat to fragment. Its efficiency, particularly in areas with dense fibrous tissue such as the male breast and back, broadened its use in such areas. On the other hand, the steep learning curve [32] and the expense of these devices [33] hindered its widespread use in low-income countries such as Egypt. We therefore sought an alternative for those patients. The idea of using the fat disruptor cannula for glandular sculpturing of the dense retroareolar tissues was attractive, as it achieved outcomes comparable to those of ultrasound-assisted liposuction with, or without, glandular excision, yet with fewer scars. The use of the fat disruptor cannula resulted in good aesthetic outcomes and was highly successful in liposculpturing of the dense glandular tissue. We presume that the barbed edges of the cannula acted as multiple microcurettes to sculpt the dense glandular tissue with minimal complications such as haematoma or prolonged neuropraxia. The differences between the fat disruptor cannula and (basket or Delvecchio cannulas) are the size and the shape of the holes at the cannula tip in addition to the shelf-like edges which facilitate fat harvesting (by means of fat disruption) and glandular tissue sculpting effect (by means of its microcuretting effect). The disadvantage of the used cannula is over sculpture of the retroareolar tissue could lead to nipple inversion which happened once in this study during the beginnings of the case series.

We had a median total operating time of 67 min and 700 ml of aspirated fat using the combined technique, which is in line with previous studies [19, 21]. We also had a shorter post-operative duration of hospital stay than previous studies [19, 21] which may be the result of the relative simplicity of liposuction that resulted in less post-operative pain and discomfort.

There is no validated assessment tool designed specifically to interpret the outcome of the treatment of gynaecomastia. The common method is to present photographs of the chest before and after operation. In our study, the preoperative and post-operative photographs were evaluated by five independent observers to strengthen the reliability of the results. Although the photographs have a standard three views, some of the observers thought it would be better to assess the patients based on a three-dimensional photograph that would simulate, to a large extent, a real clinical examination. The observers’ agreement was not strong (Kendall’s coefficient of concordance) which can be due to the lack of clear distinction for objective assessment of male breast aesthetic appearance.

### Limitations of the Study

The group of patients included was relatively small. We used suction-assisted, but not ultrasound-assisted, liposuction, which should give better results in treating the dense fibrous tissues and skin recoil, particularly in grade II gynaecomastia. Critics of ultrasound-assisted liposuction claim that the technology is expensive, requires larger incisions, and carries the risk of thermal burns [32, 34].

## Conclusion

Combined liposuction and glandular liposculpturing using the fat disruptor cannula was reliable and safe, patients were well satisfied, and the outcome was acceptable according to the observers’ ratings. It could be an alternative with a corresponding outcome to the more expensive methods.
